# Physical and Cultural Inheritance Enhance Agency, but What are the Origins of this Concern to Establish a Legacy? A Nationally-Representative Twin Study of Erikson’s Concept of Generativity

**DOI:** 10.1007/s10519-018-9943-x

**Published:** 2019-01-16

**Authors:** Kaja Faßbender, Annika Wiebe, Timothy C. Bates

**Affiliations:** Department of Psychology, University of Edinburgh, 7 George Square, Edinburgh, EH8 9JZ UK

## Abstract

Generativity—showing concern to establish and guide future generations—has been argued to be a biological adaptation central to cumulative culture and survival, but also, in turn, to be a cultural adaptation dependent on norms. From the perspective of human agency, concern for the future has played a key role in raising agency for generations that follow by creating infrastructure and cultural inheritance. Here, in a population-representative sample of 756 twin-pairs, we present the first test of the genetic and environmental structure of generativity using the Loyola Generativity Scale (short). Genetic analysis of scale sum-scores revealed that shared environmental effects were comparable in magnitude or exceeded effects estimated for genetic differences (A = 0.30 CI95 [− 0.01, 0.61], C = 0.41 [0.25, 0.56], E = 0.86 [0.79, 0.93]). At the item level, a well-fitting genetically-informed model suggested 3 factors influencing generativity via a common-pathway structure. The first was tentatively characterized as reflecting a heritable general concern for the future. The second reflected being a valued source of advice and assistance. The third factor showed only unique environment effects and had as its strongest indicator having had a good influence on the lives of others. Replicability of this structure should be tested in the full version of the scale. Work is needed also to validate influences of generativity on vocations such as teaching and on philanthropic activity improving life for subsequent generations.

## Introduction

“If I see further, it is by standing on the shoulders of giants” (Newton 1676)

“The man who dies thus rich dies disgraced.” (Carnegie 1889, p. 664)

The system of grants funded in the program represented in this special issue are focused on linking behaviour genetics with philosophy via the concept of agency. The research topics are broad in scope: from novel models of moral agency (Curry et al. 2018) to the structure of traits characteristic of high-agency individuals (Ryff 1989). In this paper, we focus on behaviors supporting future generations and the intergenerational enhancement of agency. Specifically, we examine “generativity”, defined as the desire to leave a positive legacy and accompanying activities that raise outcomes for future generations (Erikson 1963; McAdams et al. 1993). Sustaining the future has been identified as key to the welfare of future generations (Hauser et al. 2014) and legacy is an important motive for altruistic behavior (Zaval et al. 2015), but individual differences have been less well studied.

No genetically informative studies of generativity have been reported. Therefore, to better understand the structure of generativity and its environmental and genetic influences, we conducted a twin study of generativity. We motivate the study with reference to the dependence of human agency on cumulated physical and mental cultural artifacts inherited from the work of previous generations, and the norms necessary to promote such desire to leave a legacy, many of which are themselves cultural inventions (Hauser et al. 2014). We then examine the conceptual origins of the idea of generativity as a prototypically human trait, followed by an analysis of the genetic and environmental structure of individual differences in generativity.

## Agency as a cultural and genetic inheritance

The level of agency experienced by each generation is in large part dependent-upon a physical and cultural inheritance created by previous generations (Deaton 2013). This resource is provided not only by parents (Belsky et al. 2018b), but also by neighborhoods and even country (Sampson 2017). These effects operate in such diverse realms as the assurance of freedom and rule of law (Sen 1999), to uptake of education (Cowen 2011), and more abstract legacies such as science (Wootton 2015).

From the perspective of agency enhancement, the extremely large effects of these factors (Deaton 2013; Sampson 2017) raises the question: What mechanisms motivate individuals to engage in creating these legacies of infrastructure? The focus of the present paper is on the trait of generativity, identified by Erikson (1963) as key to behaviors involved in creating such a legacy.

Previous genetically-informed research on factors supporting increased agency, as manifested in outcomes such as self-control, cognitive development and educational attainment has focused on family-level factors, often with socioeconomic status (SES) as an environmental moderator (Tucker-Drob and Bates 2016; Turkheimer et al. 2003). Research capitalizing on advances in molecular genetics (Kong et al. 2018; Okbay et al. 2016) has further supported the family as a system for enhancing agency as reflected in offspring educational attainment (Bates et al. 2018). Research has also, however, been extended to examine upward social mobility (Belsky et al. 2018b) and to identify effects of the macro environment such as neighborhood on traits associated with agency, for instance obesity, mental health, teen-pregnancy, and poor educational outcomes (Belsky et al. 2018a, b).

## Beyond family: the effects of transmitted cognitive capital

As noted above many of the largest effects on individuals reflect effects of superordinate structures, with some of the largest effects existing at the level of between nation differences (Rindermann and Ceci 2009; Sampson 2017). Hunt (2012) termed these factors “*Physical cognitive artifacts*” (e.g. working sewerage and electrical systems, or computers and electronics) and “*Mental cognitive artifacts*” such as logic and formal systems of finance. Such are the benefits of such physical and mental cultural infrastructure, that cognitive adaptations specifically to support their creation have been argued to be a key element of human genetic adaptation (Lumsden and Wilson 2005). In particular, the cumulative aspect of culture—the ability to solve problems so complex that the ultimate solution must build upon partial solutions arrived at by peers—appears to be unique in humans (Dean et al. 2012).

### Generativity as an innate and socially-influenced trait

One way, then, that genetics and culture may impact agency in a given generation is by affecting the desire of individuals, rather than to consume whatever capital they have managed to build up, to instead invest this in a positive, agency-raising legacy, a motive captured in the quote from Andrew Carnegie leading this article, and reflected also in much of philanthropy. Broadly conceived, this motivation can be termed a generative urge. Erikson (1963) saw individuals as clearly motivated not only for their individual success and happiness but, especially as they aged, to focus more on giving to the common good and leaving a legacy for others. He called this motive ‘‘*concern in establishing and guiding the next generation’’* (Erikson 1963, p. 267). Erikson further viewed generativity as both an innate drive and as a culturally influenced social norm Erikson (1963).

**Fig. 1:**
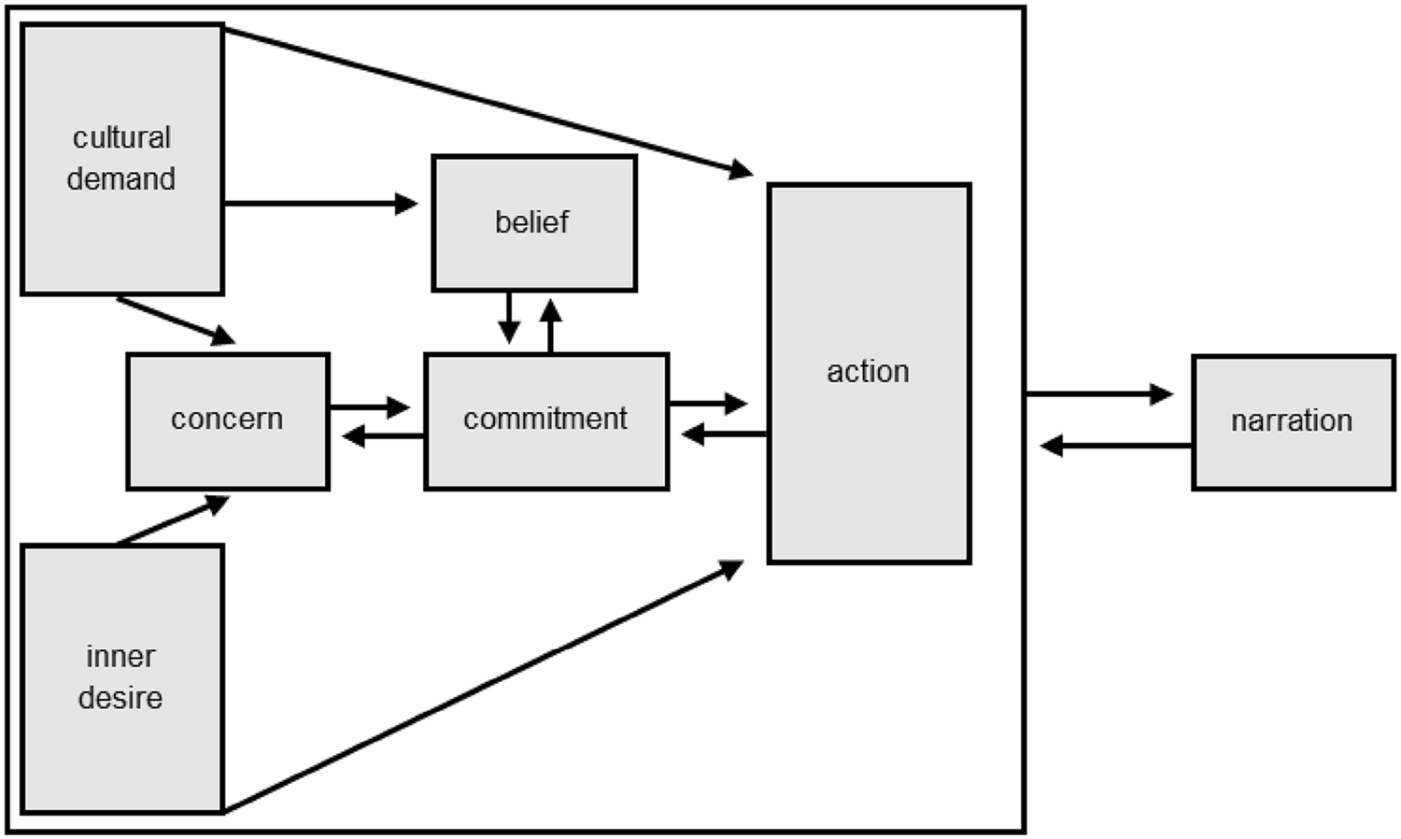
The 7-component model of generativity (Adapted from McAdams and de Aubin 1992)

Erikson embedded his idea of generativity within his larger model of psychosocial development. Since then, however, the concept has developed independently of that structure. McAdams and de St Aubin (1992) developed both a variant of generativity theory and methods for measuring the construct. These authors surveyed descriptions of generativity, synthesizing these in a model including seven social and personal elements (see Fig. 1). In their framework, generativity exists as a multi-componential potential that is latent in an individual, emerging as a more important determinant of behaviour in adulthood in response to growing opportunities and pressures to express generativity.

In the McAdams and de St Aubin (1992) model, generative behaviour results from a combination of inner desire and cultural demand, as well as feedback from generative actions. These forces provide input to an interacting internal network comprised of concern, belief, and commitment. Jointly these three elements represent a person’s feelings, values, and thoughts regarding generativity. The relation of all these elements are suggested by McAdams to be able to enter awareness as a “generative narration”, which can, in turn, influence whether and how a person pursues the goal of improving future generations.

### Measuring generativity

Earlier measures of generativity include self-ratings of several Eriksonian stages (Ochse and Plug 1986; Carol and; Heincke 1983), and assessment of components of generativity such as dominance and innovation (Carol and Migdal 1984). McAdams and de St Aubin (1992) argued that these measures correlated too highly with other constructs and lacked external validity. Responding to this perceived need, the authors constructed the Loyola Generativity Scale (LGS) to assess individual differences in generative concern (McAdams and de St Aubin 1992, p. 1006). The scales build on both existing work and their own generativity model revolving around the personality tendencies of caring about future generations (Aubin and McAdams 1995) and wishing the legacy of the self to live on (McAdams and de St Aubin 1992).

### Behavioral correlates of generativity

The LGS shows positive correlations with generative actions and narrations (McAdams and de Aubin 1992; McAdams and Guo 2015) and predicts behaviors linked to generativity including social engagement (Cox et al. 2010; Rossi 2001), authoritative parenting style and associated positive outcomes in offspring (Peterson 2006; Peterson et al. 1997), a higher level of social support from family and friends, and higher levels of religious and political engagement (Hart et al. 2001). Other research on the correlates of generativity has demonstrated links to well-being and life satisfaction (Ackerman et al. 2000; An and Cooney 2006; Aubin and McAdams 1995; Cox et al. 2010; Grossbaum and Bates 2002; Keyes and Ryff 1998; McAdams et al. 1993). While more work is needed to demonstrate if high levels of generativity affect, for instance, innovations improving social, political, or commercial life, and choices made which improve outcomes for future generations, it is fair to say that the LGS appears to be a valid measure according to evidence from multiple realms: from family and relationships to work and society and encompasses new creations that help future generations as well as direct guidance of future generations (McAdams 2013).

Regarding the developmental course of generativity, Einolf (2014) reported that generativity is relatively stable (> 0.60 over a 10-year period), but also that it attains near-adult levels at least from the mid-20s onward, if anything showing a mid-life peak. Interestingly, marriage and childbearing were not associated with an increase in LGS scores, suggesting that this trait is, as theorized linked to a drive to support future generations more broadly rather than being a simple reflection or extension of family life (Einolf 2014).

### Why a twin study of generativity?

Despite its relevance for the individual and the society, relatively little research has addressed the structure and origins of generativity, and none (that we are aware of) has examined the heritability of generativity. In review, we were asked why is a twin study still important? There are several reasons. Generativity theory claims a relatively strong influence of shared environment on generativity (Erikson 1963; McAdams and de Aubin (1992; McAdams and Guo 2015; Rossi 2001). This would make it unusual among most traits in adulthood, where shared environment (“C”) is usually modest in magnitude and relative to genetic effects (Turkheimer 2000). For instance adult cognitive ability has been reported as showing near-zero levels of shared environmental influence in adults (Deary et al. 2006). The prediction from generativity theory of a substantial C cannot be falsified, however, by appeal to a common pattern—it must be tested. It is therefore important to test the prediction that shared environment will play a large role, perhaps comparable too, or even larger than that of genetic differences in generativity. There are also violations of this pattern in other traits. A number of abnormal traits, for instance, show relatively high levels of shared environmental influence that endure into adulthood (Burt 2009). In addition, though less often studied by behavior geneticists, attitudes are emerging as a class of traits with relatively weak genetic influence. While genes appear to be responsible for stability in some attitudes (Lewis and Bates 2017), such measures often show equivocal evidence for genetic and shared environmental influences (Lewis and Bates 2011; Martin et al. 1986). Generativity, then, may show influences of genes that are uncharacteristically low compared, for instance to genetic influences on cognitive abilities (Engelhardt et al. 2015), along with uncharacteristically elevated levels of shared environmental influence in adulthood.

A widely accepted reason to document the magnitude of variance attributable to genetic, family-environment, and unshared environmental influences, not just in a given sample (which is of limited value), but in many samples. This impetuous flows from the finding that variance attributable to genes and environment can not only take on different values depending factors such as age or birth cohort (Briley et al. 2015), but may vary quantitatively and qualitatively across sex (Neale and Maes 1996), and can be moderated by family-level factors such as socioeconomic status (Turkheimer et al. 2003) or by linked factors such parenting style—a classic example being the suppression of genetic influences on disinhibition by parental religiosity (Boomsma et al. 1999).

Understanding and incorporating these moderators such as culture, country, cohort into causal, mechanistic psychological theories requires, at least in the first instance, collecting evidence for their existence and dozens of well-powered studies across representative levels of each factor to map the space with some modicum of precision. Currently large regions of the world have no substantial twin studies even of core traits such as cognitive ability—India, Africa, and China for instance—while many specific domains of behavior or attitudes remain to be studied even once.

While a complete theory of behavior genetics should be able to explain why traits show the levels of genetic or shared environmental influence observed, we are not in a position to do this, in part because the data required (estimates of genetic, and shared environmental variance at different levels of proposed moderators, in different societies, and at different ages, and across sexes) have not been collected. It is of value, then, to collect data on generativity, and to establish whether it has high, relatively modest, or even low levels of heritability, not only to add to the rather sparse database of heritabilities in the domain of attitudes and motives, but to begin to document variation in these traits across cultures and times.

A further reason to examine generativity in a genetically informative sample is that in this, as in all areas of social science, concrete estimates of genetic influence not only to test theory that includes such influences, but also motivate other research to include controls for genetics. As was noted in a recent commentary, even longitudinal relationships of parent and offspring behavior are not evidence for effects of parental behavior (Sherlock and Zietsch 2018). Documenting genetic and familial influences are important factors influencing the design of studies in which genetics would otherwise confound the causal logic of developmental studies. At present, no estimate of heritability for generativity exists to provide such a concrete stimulus.

A final motivation for studying the item-level heritability of generativity is that the genetic and environmental homogeneity (or heterogeneity) of generativity items has not been examined. While the phenotypic null hypothesis (Loehlin and Martin 2013; Turkheimer et al. 2014) often holds, it is a null hypothesis. That is, we cannot state with certainty that a given measure will reveal genetic, shared and unique environmental structures identical to that of its phenotypic structure. Instead, it is possible for genetic and shared environmental influences to diverge from the phenotypic factor structure qualitatively as well as quantitatively. As an example, work on optimism–pessimism (Scheier et al. 1994) has often treated these as a single bi-polar dimension, even as just a reflection of emotional-stability (Sharpe et al. 2011). However, under genetic analysis, orthogonal genetic influences on optimism and on pessimism have been revealed, along with a complex family-environmental structure (and no genetic factors) linking optimism and pessimism to neuroticism (Bates 2015).

Related to this task of decomposing the phenotype, twin studies can test the neurobiological coherence of a construct. Kendler (2013) gives the humorous example of an imaginary syndrome called “LLR”, comprised of left handedness, long nose and red hair. Diagnosis for LLR syndrome would appear to be familial and (modestly) heritable, perhaps leading to a claim that LLR reflects the action of a unitary biological system. The claim that LLR behaviors are biological, however clearly does not, in itself, warrant the coherence of the category at a biological level. A twin study of the three LLR “symptoms” would reveal that in fact these are genetically unrelated—that is that the genetic program responsible for the biology of each symptom of the supposed syndrome lies on segments of DNA which segregate independently. While molecular studies with a hundred thousand subjects are now powered to detect item-level structure and cross-disorder and even cross-sample GWAS summary statistics (Grotzinger et al. 2018), twin studies provide a well-powered tool to undertake the same tasks of testing biological and environmental coherence (Kendler et al. 2013) and even direction of causation (Gillespie et al. 2012). There are several reasons, then to test the scale and item-structure of generativity in a twin study. We next move to the aims and hypotheses of the present study.

## The present study

Our aim in the present study was to test the behavior genetic structure of the generativity scale, capitalizing on twin data to distinguish influences of genes, and of shared and unique environments. The classic twin design decomposes variance into additive genetic effects (A), shared or common environmental influences (C) shared by both twins, e.g. place of residence or neighborhood factors, and unique environment (E): environment influences which are not shared, e.g. their unique peer-group (Neale and Maes 1996).

In hypothesizing what structure of A, C, and E one should predict to account for generativity sum-scores, we were informed by Erikson’s own (1963) theoretical structure described as involving both innate and cultural influences. Existing data on parent-offspring correlations of around 0.4 between parents’ and children’s generativity scores (Peterson 2006) confound shared environment and genetic influences, but imply that one or both of these factors should show significant effects. Rossi (2001) has also hypothesized that both genetics and parental socialization should influence generativity. Analyzing personality traits and parental characteristics correlated with offspring generativity in the MIDUS he showed that the personality traits of agency and communion (which he took as strong predictors of adult generativity), had a heredity component of 42 and 46%, respectively, implying a role for genes in generativity, and assuming a role for shared environment.

Following McAdams and de St Aubin (1992) and Erikson (1963), we therefore predicted, that LGS scale-scores would show significant effects of both additive genetic and of shared environmental influences (as well, of course, as unique environmental effects, which include measurement error).

### Item-level factor structure

In terms of the item-level factor structure of the LGS (short), while the scale is designed to be unifactorial, we were open minded as to the factor structure that would be revealed in a genetic analysis. In particular, the multiple components and influences on generativity identified in modern generativity theory (Aubin and McAdams 1995; McAdams and de Aubin 1992; McAdams and Guo 2015) and shown in Fig. 1 suggest multiple distinct elements, with distinct environmental and genetic influences (or even no genetic influence in the case of factors external to the family) may play a role in manifested generativity. If the items of the scale differ in their genetic and environmental architecture, then an item-level analysis of the scale may be able to decompose these distinct influences.

Based on Ockham’s razor and also the previous factor-analytic evidence, we predicted that a common pathway model (see Fig. 3 and analysis plan below) with a single common factor would account adequately for variance in the generativity items, again with significant genetic and shared environment input to this common factor. Because McAdams and de St Aubin (1992) identify multiple distinct causal elements underpinning generativity, we predicted that if multiple factors were required to fit the behavior genetic data, these would conform to causal roles from one or more of the modules of the 7-factor generativity theory—for instance cultural demand could raise variance in some or all aspects of generative action, presumably through either shared, or unshared environmental routes. Similarly, individual differences in inner desire, concern for the future, trait levels of commitment, incorporation of cultural and self-originated commitment into beliefs might fall out as distinct factors. The ability of the common pathway analysis to model such multi-factorial structure (and the less constrained biometric or independent pathway model) is described below.

#### Data analysis plan

**Fig. 2:**
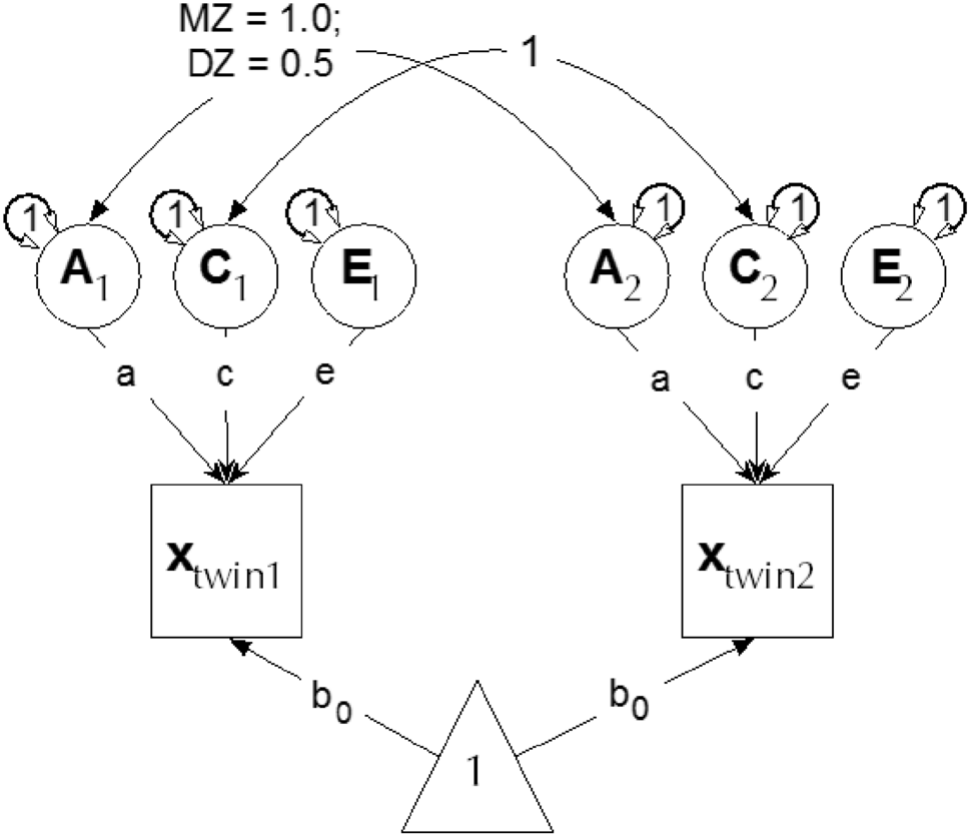
Univariate Cholesky decomposition. The model shows latent additive genetic (*A*), shared environment (*C*) and unique environment (*E*) influences, and their paths (a, c, and e) to the manifest (measured) trait “x” for twin 1 and twin 2 of each twin pair. Curved paths show the covariance of *C* (1.0 in both MZ and DZ groups) and how the covariance between *A* latents takes the value 1.0 in the MZ group, and 0.5 in DZ group. The means of the data are modelled via b_0_ paths

For the univariate analysis of generativity scale-scores, we used the classic ACE decomposition (Neale and Maes 1996), which permits the estimation of an additive genetic factor (A), either dominance (D) or shared environmental factor (C), and a third “E” factor accounting for unique environment variance (see Fig. 2). Readers should note that the classic twin model is predicated on testable assumptions such as equal environments across zygosity (Kendler et al. 1993) and assortative mating (Swagerman et al. 2017). Other aspects of the models, like all models, depend on appropriate modelling to avoid mis-allocating variance (Keller and Coventry 2005; Turkheimer and Waldron 2000). Important among these are assumptions about the presence of unmodeled interactions. For this reason, we also undertook an examination of potential gene-environment and environment–environment moderation of A, C, and E effects by factors such as socioeconomic status (Rowe 2001; Tucker-Drob and Bates 2016; Turkheimer 2016). This was done using univariate interaction models specified in Purcell (2002).

**Fig. 3:**
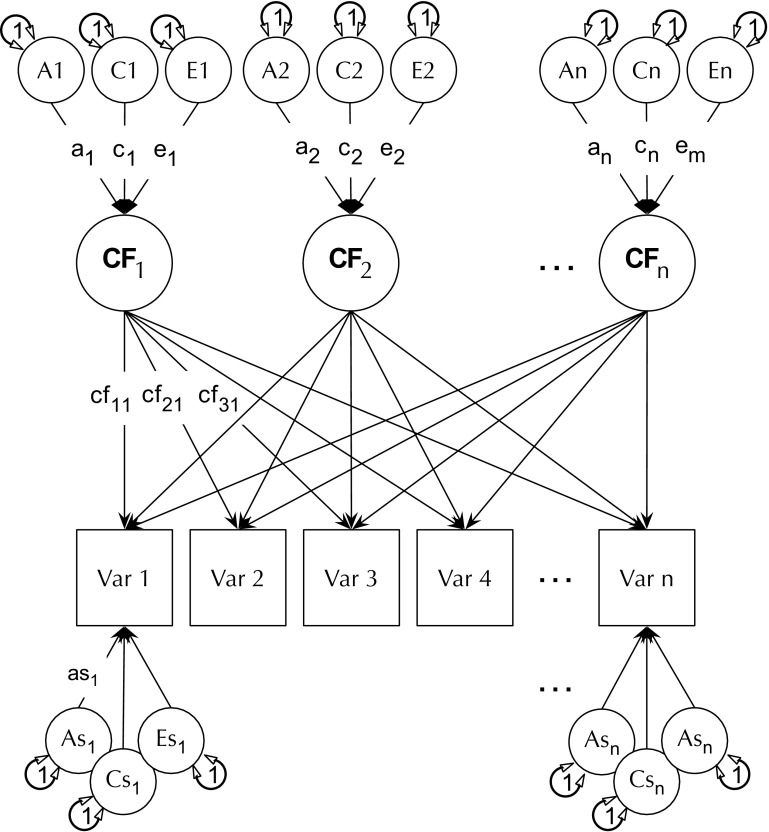
Example Common Pathway Model. “A”, “C”, and “E” are additive genetic, common environmental, and unique environmental latent factors, respectively. Latent “as”, “cs”, and “es” are additive genetic, common environmental, and unique environmental specific influences, respectively

For the multivariate item-level analyses, our plan included using a common pathway model and also independent pathway models of the variance, representing different ways in which genes and environment might account for variance and covariance in these individual items, including allowing for multiple factors among measured items (Neale and Maes 1996). A common pathway model (CPM) is shown in Fig. 3. This model assumes that gene and environmental factors contribute to a latent psychometric factor, which then in turn is manifested in the measured variables. In other words, the genetic and environmental influences act on items via a common pathway, hence the name. In the second type of theoretical model used—the independent pathways model (IPM)—no common psychometric latent traits are postulated. Instead, latent genes and environments factors act directly on each item, potentially having different effects on the pattern of item covariation. Both the CPM and IPM models can be extended to have more than one A, C, and E factor, thus modelling more complex patterns of covariance among the measured variables (Neale and Maes 1996). The CPM is subsumed within the IPM, and fit of the two models can therefore be compared using a likelihood ratio χ^2^ test.

### Model fit and choice of a baseline model

**Fig. 4:**
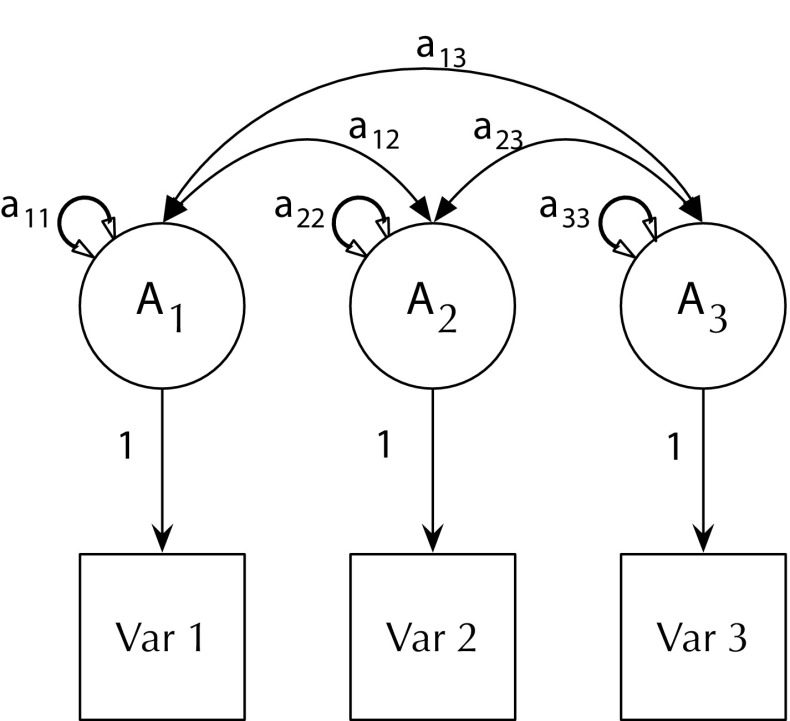
Baseline Direct variance–covariance ACE decomposition. For clarity, showing only Twin 1 variables and also omitting paths from the C and E matrices. Each measured item has its own genetic (and common environmental, and unique environmental latent factors). These have estimated variance (e.g. a_11_) and can covary with the variance components for the other manifest variables. Variance of each manifest and covariances among these is thus estimated directly as the sum of A, C, and E influences

Model fit can be statistically evaluated by means of a likelihood comparison against a baseline model. For the univariate model, the saturated model has no degrees of freedom and thus serves as its own baseline for tests, for instance, of the significance of individual paths. For multivariate models (in this case item-level models) the choice of a baseline model is more complex. While it is common practice to evaluate such models against a multivariate Cholesky ACE model, algebraically, this model is not unbiased (Carey 2005). For this reason, we used instead the theoretically preferable direct variance–covariance ACE model (see Fig. 4). As in the Cholesky model, the direct variance–covariance model decomposes the variance of each manifest variable into additive genetic (A), common environmental (C) and unique environmental (E) components. However, unlike the Cholesky model (which takes its name from its use of intermediate matrices comprising a Cholesky factor decomposition of path coefficients which are post multiplied by their transpose to estimate the manifest variance covariance matrix) the direct variance–covariance decomposition directly estimates the variance attributable to the A C and E variance components. This achieves a saturated and unbiased model (Carey 2005; Verhulst et al. under review).

## Methods

### Participants

The data for this study originate from Wave II of the MacArthur Foundation Survey for Midlife Development in the US (MIDUS: Brim et al. 2004) containing data from 851 twin pairs of a nationally representative sample of households. Our sample consists of 856 female (mean age 53.96 years, age range 34–84, SD = 11.86) and 656 male (mean age 54.36 years, age range 34–82, SD = 11.37) twins where at least one twin in a pair had completed the generativity questionnaire. A total of 756 complete twin pairs were represented. Of these, 278 were monozygotic (153 female and 125 male) and 478 dizygotic (175 were female, 103 male and 200 opposite sex).

## Measures

### The Loyala generativity scale-short (LGS-Short)

We used an abbreviated and modified version of the 20-item Loyala Generativity scale, developed for MIDUS (see Appendix 2). The short scale consists of 6 items (α = 0.85), some of which match items from the original scale, while others have been reworded in an attempt to cover multiple elements of generativity. The short scale has been reported as fitting a one factor structure (Einolf 2014). We confirmed this with a parallel analysis (Horn 1965) indicating clear support for a 1-factor solution, with only one factor showing an adjusted eigenvalue greater than expected by chance (adjusted eigenvalue for factor 1 = 3.03). A maximum likelihood factor analysis indicated this factor accounted for 50.5% of variance in the scale, with item loadings ranging from 0.59 to 0.776. Items include statements like “*Others would say that you have made unique contributions to society*” or “*You like to teach things to people*”. The scale showed acceptable reliability (Cronbach’s α = 0.85).

Despite considerable overlap, the long and short scales differ in a number of substantive ways. The items are written in the third-person, and all are positively worded, whereas in the full version, items are in the first-person tense and include negative items (e.g. “*I have done nothing of worth to contribute to others*”). The long scale is explicitly focused of effort and success at passing along knowledge (e.g. “*I have made a difference to many people*”) where the short scale focusses on possessing such knowledge or skills. The long scale also measures the enjoyment of a vocation (such as being a teacher), as opposed to “*liking to teach things to people*” in the short scale. Some behaviors are absent, for instance volunteering for charity, creative effort, commitment, productivity, neighborhood responsibility. The full version also emphasizes the creation of a legacy that will be remembered beyond a person’s own lifetime.

Respondents rated on a 4-point Likert scale ranging from 1 (‘A lot’) to 4 (‘Not at all’).

## Statistical analyses

### Univariate twin modelling

To test the hypothesis that familial clustering in generativity is explained by both additive genetic and family environment factors (in addition to unique environmental influences including measurement error), we fitted univariate biometrical genetic models (Neale and Cardon 1992). Based on the Classical Twin Design (Jinks and Fulker 1970; Neale and Cardon 1992), our univariate modelling used the expected genetic and environmental correlations between monozygotic (MZ) and dizygotic (DZ) twins to decompose individual differences in generativity into additive (A) genetic, shared environmental (C), and non-shared or unique (E). We fitted twin models using the FIML (Full Information, Maximum Likelihood) capability of the OpenMx package (Neale et al. 2016) in R 3.5.1 (R Core Team 2017). Since MZ twin pairs are genetically identical and DZ twin pairs share, on average, half of their genes, the expected twin pair correlations for additive genetic effects are 1.0 and 0.5 respectively. An important assumption is that the common environments (C) are equal in MZ and DZ twin pairs, and that assortative mating is negligible although this can also be modelled by fitting differing values for the DZ additive genetic correlation (Loehlin et al. 2009). Because non-shared environments (E) are uncorrelated, E necessarily includes measurement error. All data were residualized for covariates of age and sex.

### Multivariate twin modelling

To test the hypothesis that genetic factors in each of the generativity items reflect a single common continuum of liability (and later to test the possibility that more than one factor is expressed), we again used the OpenMx package (Neale et al. 2016) and R (R Core Team 2017) to fit common- and independent-pathway models (Neale and Cardon 1992), beginning by fitting a single CPM to the 6 generativity items. Each model was tested by comparing its fit to that of a saturated variance-components model (Verhulst et al., under review). As illustrated in Fig. 3, the CPM assumes that there exist one or more common latent pathways to generativity, each of which reflects additive genetic (A), shared environmental (C), and non-shared environmental (E) components. The figure shows how each common pathway is indicated by the manifest or observed phenotypic measures (generativity items). Variance unique to each item is decomposed into component-specific elements of variance: as; cs; and es.

We began by fitting a model with a single psychometric common pathway, with all parameters estimated. Following this, we tested fit, and, as proved necessary, tested an IPM, as well as CP and IP models with 2 or 3 factors. We subsequently tested models in which all ‘C’ parameters were fixed to zero, and in which all ‘A’ factors were set zero. Fit was assessed using the Akaike Information Criterion (AIC: Akaike 1974) which balances complexity against explanatory power. AIC differences among competing models were presented also in terms of AIC weight-based conditional probabilities to facilitate interpretation (Wagenmakers and Farrell 2004). These were computed using the R package *MuMIn* “Weights” function with model AICs as input.

## Results

### Descriptive statistics

Table 1 shows the mean scores and standard deviations of each of the six generativity items as well as the correlations (and SEs) for the MZ and DZ groups (one subject from each pair used) for each item. Mean levels suggested that most people are low to moderately concerned about the care and future of the next generation. At an item level, generativity was correlated weakly across the twins with modest increments in association for the MZ versus the DZ twin pairs. The DZ twin pair correlation was typically more than one-half of the MZ counterpart, which is consistent with the hypothesis that familial clustering is significantly influenced by shared environment factors.

**Table 1:** Mean scores and standard deviations, on the individual items, separately for twin1 and twin2 in each group [monozygotic (MZ) and dizygotic (DZ)] of twins, and the correlations between twin 1 and twin 2 for each item, separately by group

	MZ				DZ				Twin 1–twin 2 correlation (SE)	
	T1		T2		T1		T2			
	M	SD	M	SD	M	SD	M	SD	MZ	DZ
Item 1	2.51	0.92	2.55	0.95	2.45	0.91	2.48	0.88	0.20 (0.08)	0.19 (0.06)
Item 2	2.17	0.86	2.15	0.80	2.14	0.82	2.06	0.80	0.28 (0.07)	0.15 (0.06)
Item 3	2.30	0.86	2.23	0.76	2.25	0.81	2.23	0.78	0.29 (0.07)	0.16 (0.06)
Item 4	2.17	0.84	2.23	0.79	2.18	0.78	2.08	0.87	0.20 (0.08)	0.18 (0.06)
Item 5	2.04	0.85	2.08	0.78	2.08	0.78	2.01	0.82	0.10 (0.08)	0.13 (0.06)
Item 6	1.92	0.89	1.91	0.83	1.86	0.81	1.89	0.83	0.16 (0.08)	0.10 (0.06)
Scale mean	2.19	0.69	2.19	0.63	2.16	0.60	2.12	0.63	0.28 (0.07)	0.20 (0.06)
Average for single items									**0.21**	**0.15**

### Univariate results

**Fig. 5:**
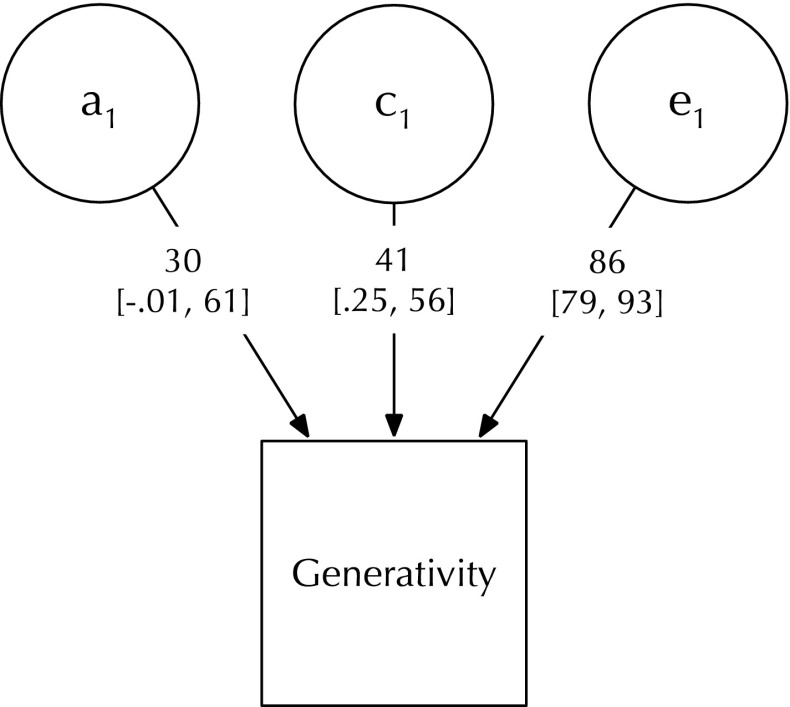
Univariate ACE analysis of Generativity scale-scores showing path coefficients (95% CIs in brackets). For clarity, decimal places not printed on parameter estimates

The standardized variance components for the full univariate model generativity scale-scores are shown in Fig. 5, and the fit statistics for models dropping C and dropping A from the model are presented in Table 2. AIC weight-based (Wagenmakers and Farrell 2004) conditional probabilities for the ACE, CE and AE models respectively were: 0.20 0.50, and 0.30, suggesting a slight preferability of a CE model over either the AE or ACE models. As both the AE and CE models provided equivocal fit to the data, we show the unreduced ACE model (see Fig. 5).

**Table 2:** Fit statistics for univariate ACE, CE and AE models of generativity scale-scores

	Model	EP	Δ − 2LL	Δ df	p	AIC	Compare with model
1	ACE	4.00				− 102.00	
3	CE	3.00	0.24	1	0.628	− 103.76	ACE
4	AE	3.00	1.27	1	0.259	− 102.73	ACE

Lower AIC is better, p-value reflects the significance of dropping A or C from the ACE baseline model

In the saturated model, additive genetic risk factors explained 8.8% of the total variation. Relative to many traits studied, this level of genetic influence is relatively small. Shared environmental factors accounted for approximately twice this proportion of variance in generativity (16%). The remaining proportion of variance was explained by non-shared or stochastic environmental risk factors including measurement error.

In an exploratory modelling phase, we also ran a gene × environment interaction model (Purcell 2002) testing if gene or environment variance might be moderated by the childhood socioeconomic environment (SES) experienced by the subjects, as is the case for their attained cognitive ability (Bates et al. 2013). There was, however, no support for moderation by SES of A, C, or E variance and all three moderation paths could be removed without significant loss of fit (χ^2^ (3) = 1.61, p = 0.657) and an improvement of AIC compared to a model containing these paths (AIC = − 96.00 versus − 91.6 including moderation).

### Multivariate item-level behavior genetic analyses

We next turned to the item-level data, to test our hypothesis that, under behavior genetic analysis, the items of the scale would be well explained by a single common factor model with significant genetic (A) and shared environment (C) influences (as well as unique environment, E). To this end, we computed a 1-factor CPM, and compared this to the direct variance–covariance baseline model (see Table 3 for fit comparisons).

**Table 3:** Comparison of item-level models of generativity: comparing 1, 2, and 3-common pathway (CP) and independent pathway (IP) models with the baseline (saturated variance–covariance model)

Model	EP	∆ − 2LL	∆ df	p-value	AIC	Compare with model
Baseline	69				607.67	
1-factor CP	33	136.77	37	< 0.001	678.96	Baseline
2-factor CP	42	49.37	29	0.011	607.56	Baseline
3-factor CP	**51**	**9.85**	**21**	**0.981**	**584.05**	Baseline
1-factor IP	42	68.08	27	< 0.001	621.75	Baseline
2-factor IP	60	13.99	9	0. 123	603.65	Baseline
3-factor CP	51	4.42	12	0.974	584.05	2-factor IP
3-factor CP (no C)	42	2.10	9	0.999	568.18	3-factor CP
3-factor CP (no A)	42	3.25	9	0.999	569.33	3-factor CP

The best-supported (according to AIC) un-reduced model (3-factor CP model) of generativity shown in bold

As can be seen in Table 3, a 1-factor CPM fit poorly compared to the direct variance–covariance baseline model (χ^2^ (37) = 136.77, p < 0.001). We then explored sequentially more-complex models. A 1-factor IPM also showed inadequate fit (p < 0.001). We then executed CPM and IPM models with 2 and 3 factors, finding that a 3-factor CPM model fit well (χ^2^ (21) = 9.85, p < 0.98, see Table 3). A 2 factor IPM also did not fit significantly worse than the baseline model (χ^2^ (9) = 13.99, p < 0.123) but was not preferred by AIC compared to the 3 common pathway model (AIC for the 3-factor CPM = 584.05 versus 603.65 for the 2-factor IPM, see also Table 3). This complexity was unexpected.

### Tentative model reduction

**Fig. 6:**
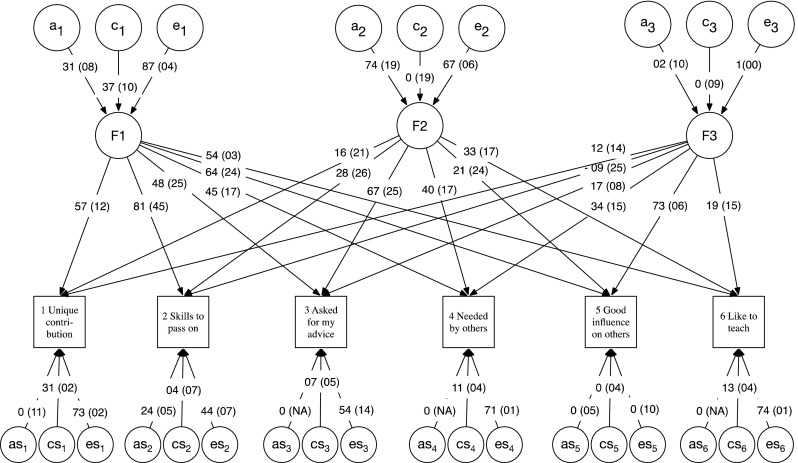
Final 3-factor common pathway model of generativity. F1-F3 are the three psychometric factors of the model. “A” “C” and “E” are common latent genetic shared- and unique-environmental influences respectively, and “as” “cs”, and “es” the corresponding specific influences (SE in brackets). For clarity, decimal places not printed on parameter estimates

Given the modest power of the present study, we do not report an attempt to remove all non-significant paths, and instead report the full model with confidence intervals (see Fig. 6). We did evaluate the relative merit of 3-factor CPM models in which all C influences (AE model) were dropped compared to a model in which all A influences (CE model). Neither model incurred a significant loss of fit (p = 0.999, see Table 3), indicating equivocal evidence for the source of familial clustering for generativity.

## Discussion

The univariate results supported the hypothesis that generative concern is subject to substantial shared-environmental effects, such that in this dataset, evidence for C effects exceeded that for additive genetic influences, which were modest and smaller in magnitude. By contrast, our item-level hypothesis (that a 1-factor common pathway model would fit well) was disconfirmed. Instead, the analyses revealed a complex factor structure best accounted for in terms of three psychometric factors in a common pathway model. We discuss these two findings in more detail below.

A surprising result of genetic research has been that often (Plomin 2011), but not always (Bates 2015; Burt 2009), behavior genetic data indicated absence of significant shared environmental effects. Following McAdams and de St Aubin (1992) and Erikson (1963), we predicted that for generativity, parental socialization would play a role, and, therefore, that shared environment effects would be significant in these analyses. Shared-environment refers to all non-genetic factors that differentiate between families and make twins more similar to each other, for example socio-economic status of the family, the neighborhood they live in or parenting style (Neale and Maes 1996). This result, then, supports a role of environmental effects aggregating at the familial level. Unshared environmental effects were significantly larger again. This result is compatible with the role of differential exposure to cultural norms and systems which are supportive of positive attitudes toward the future and leaving a legacy to future generations via mechanisms discussed, for example, by Hauser et al. (2014) and Janssen et al. (2010) and related to producing social systems reinforcing and communicating norms regarding these attitudes.

The results also suggest that an expectation of zero *C* effects, for all behavior genetic traits, may be premature, not only because of GE covariance in different forms (Bates et al. 2018; Plomin 1994; Turkheimer and Waldron 2000), but also for simply a lack of power to detect C relative to A (Neale and Maes 1996), ability to capitalize on aggressive dropping of blocks of paths masking significant effects within these large blocks, and inherent biases of the Cholesky model as a baseline against which to test significance of the shared environment (Verhulst et al., under review).

### Multivariate item-level analyses

The analyses supported three independent common factors accounting for variance in the short LGS. This was interesting both in virtue of the more complex structure compared to that seen in purely phenotypic analyses (Einolf 2014), and, of course, for the psychological meaning that may be attributed to these three factors. Divergence between phenotypic and behaviour genetic structure is uncommon—genetic studies mostly are not expected to uncover deeper structure (Turkheimer 2016). As Loehlin and Martin (2013, p. 761) put it with respect to personality “*the structure…is inherent in the evolved phenotype*” rather than being organized by immediate genetic or environmental factors. The present findings diverge from this pattern, reflecting differential patterning among the items. The result supports the role for genetically informative samples in sub-typing distinctly segregating components of behavior, and for arriving at meaningful distinctions in the origins of these components in terms of their dependence on purely environmental effects, or combinations of genetic and environmental forces. This approach has been successful in psychiatric genetics (Kendler et al. 2013). For instance, behavior genetic decomposition of Autism symptoms indicates that this is not a unitary syndrome, but that a diagnosis likely reflects three independent diseases (Happe and Ronald 2008; Happe et al. 2006).

One potential explanation for the structure is that rather than measuring purely the “concern” component of McAdams et al. (1993) 7-component model of generativity (see Fig. 1), the short scale captures additional aspects of this structure. We next turn to the meaning of the three factors which emerged, bearing in mind possible links to this larger framework.

Factor 1, loaded above 0.45 on all 6 items. It thus behaves as predicted for the “generative concern” component of McAdams et al. (1993) theory. This factor showed high levels of unique-environmental variance, and roughly equal influences of genes and shared environment. The highest loading item was having skills to pass on (0.81). Factor two showed strong loadings on only two items: being asked for advice and being needed by others. The third highest loading was liking to teach. Other loadings were, in this sample, non-significant. This factor showed the highest influence of genes and negligible evidence of shared environment influence. Factor 3 was almost entirely reflective of unique environment variance. The strongest loading was *“You have had a good influence on the lives of many people”*. The next highest loading items were being needed by others and enjoying teaching. Reflecting unique environments, this factor may tap the cultural-demand component of McAdams’s model, or perhaps opportunities which generate outsized but unpredictable increments in the ability to leave a positive legacy for an individual, somewhat analogous to the out-size influence of initially similar individuals ending up with very different outcomes in Pareto-type systems of reward.

If we take factor 1 as tapping internalized generative concern, the environmental factor 3 as tapping the unique experience of environmental opportunity and cultural demand, then, within the 7-component model, a plausible, though speculative, candidate for factor 2 would be inner-desire, in keeping with reported pleasure directly in the acts of caring and teaching. The three distinct pathways emerging here, then, may reflect both general level of concern and two inputs to that concern: inner desire and exposure to cultural pressure.

## Limitations and future study

The study has limitations. Given power of the present study to resolve competing models (for instance the 2 independent pathway and 3 factor common pathway models did not differ greatly according to AIC), it would be valuable to repeat the analyses with the full scale, and, preferably, in a larger sample. We are undertaking this task as part of the project this year.

It would be of value also to have measures for more of the 7 predicted components of generativity, work that would not require access to a twin sample. Given the importance attributable to education in modern society, it would seem useful to understand better the origins of motivation to teach. Based on low rates of systematic teaching (as opposed to allowing children to watch skilled tasks, or practice these themselves) in pre-industrial society, Lancy (2016) suggests that while children are adapted to copy and learn, adults are not adapted to explicitly teach. The present data suggest at least some heritable influence on interest in teaching. It would be valuable to explore such motives in more depth.

The present sample was predominantly middle aged. It would be valuable to study generativity in younger subjects (to understand its developmental origins). Also of value would be to measure wider social effects of generativity on important prosocial activities such as institution founding and maintenance.

## Summary

With these limitations, this study is the first step to understanding the genetic and environmental structure of generativity. It demonstrated that, at a scale level, generativity reflects large influences of the environment, including shared environment, while revealing a more complex structure at the item level.

## Appendices

### Appendix 1: Loyola generativity scale

1. I try to pass along the knowledge I have gained through my experiences.
2. I do not feel that other people need me.
3. I think I would like the work of a teacher.
4. I feel as though I have made a difference to many people.
5. I do not volunteer to work for a charity.
6. I have made and created things that have had an impact on other people.
7. I try to be creative in most things that I do.
8. I think that I will be remembered for a long time after I die.
9. I believe that society cannot be responsible for providing food and shelter for all homeless people.
10. Others would say that I have made unique contributions to society.
11. If I were unable to have children of my own, I would like to adopt children.
12. I have important skills that I try to teach others.
13. I feel that I have done nothing that will survive after I die.
14. In general, my actions do not have a positive effect on other people.
15. I feel as though I have done nothing of worth to contribute to others.
16. I have made many commitments to many different kinds of people, groups, and activities in my life.
17. Other people say that I am a very productive person.
18. I have a responsibility to improve the neighborhood in which I live.
19. People come to me for advice.
20. I feel as though my contributions will exist after I die.

### Appendix 2: Reduced Loyola generativity scale (from MIDUS)

1. Others would say that you have made unique contributions to society.
2. You have important skills you can pass along to others.
3. Many people come to you for advice.
4. You feel that other people need you.
5. You have had a good influence on the lives of many people.
6. You like to teach things to people.
